# Variation in ampicillin dosing for lower respiratory tract infections and neonatal bacterial infections in US children’s hospitals

**DOI:** 10.1017/ash.2022.221

**Published:** 2022-05-23

**Authors:** Elizabeth A. Daniels, Christopher C. McPherson, Jason G. Newland, Brian R. Lee

**Affiliations:** 1 Department of Pediatrics, Washington University School of Medicine, St. Louis, MO; 2 Department of Pediatrics, University of Missouri-Kansas City School of Medicine, Kansas City, MO

## Abstract

**Objective:**

We examined ampicillin dosing in pediatric patients across 3 conditions: (1) bacterial lower respiratory tract infections (LRTIs) in infants and children >3 months, (2) neonates with suspected or proven sepsis, and (3) neonates with suspected central nervous system (CNS) infections. We compared our findings to dosing guidance for these specific indications.

**Design:**

Retrospective cohort study.

**Setting:**

The study included data from 32 children’s hospitals in the United States.

**Methods:**

We reviewed prescriptions from the SHARPS study of antimicrobials, a survey of antibiotic prescribing from July 2016 to December 2017. Prescriptions were analyzed for indication, total daily dose per kilogram, and presence of antimicrobial stewardship program (ASP) review. LRTI prescriptions were compared to IDSA recommendations for community-acquired pneumonia. Neonatal prescriptions were compared to recommendations from the American Academy of Pediatrics (AAP). Prescriptions were categorized as “optimal” (80%–120% of recommended dosing), “suboptimal” (<80% of recommended dosing), or “excessive” (>120% of recommended dosing).

**Results:**

Among 1,038 ampicillin prescriptions, we analyzed 88 prescriptions for LRTI, 499 prescriptions for neonatal sepsis, and 27 prescriptions for neonatal CNS infection. Of the LRTI prescriptions, 77.3%were optimal. Of prescriptions for neonatal sepsis, 81.6% were excessive compared to AAP bacteremia recommendations but 78.8% were suboptimal compared to AAP meningitis guidelines. Also, 48.1% of prescriptions for neonatal CNS infection were suboptimal, and 50.6% of prescriptions were not reviewed by the ASP.

**Conclusions:**

LRTI dosing is generally within the IDSA-recommended range. However, dosing for neonatal sepsis often exceeds the recommendation for bacteremia but is below the recommendation for meningitis. This variability points to an important opportunity for future antimicrobial stewardship efforts.

Ampicillin, a third-generation penicillin antibiotic, has an excellent safety profile and low cost. In infants and children, it is a preferred therapy for uncomplicated, community-acquired pneumonia (CAP)^1^ and for the empiric treatment of neonatal sepsis and meningitis. In a recent point-prevalence study of antimicrobial use at US children’s hospitals, ampicillin was the seventh most commonly prescribed antibiotic;^2^ it is also one of the most frequency prescribed medications in neonatal intensive care units (NICUs) worldwide.^3^

However, despite frequent use, recommendations for ampicillin dosing vary widely, with general dosing recommendations ranging from 50 to 400 mg/kg/day. For empiric treatment of lower respiratory tract infections (LRTIs), such as CAP, the dosing guidance is relatively clear; the Infectious Disease Society of America (IDSA) guidelines suggest an empiric treatment range of 150–200 mg/kg/day, except in cases of known resistance.^1^ In contrast, dosing guidelines for neonatal bacteremia and meningitis differ between commonly used sources, including Lexicomp, Neofax, *The Harriet Lane Handbook,* and the *Nelson Textbook of Pediatrics*, which are based upon variable combinations of weight, gestational age at birth, corrected gestational age, and postnatal age.^4–7^

In 2014, dosing recommendations emerged based on pharmacokinetic data in the neonatal population, developed by the Understudied Drugs Administered to Children per Standard of Care Study (POPS), a National Institutes of Health-sponsored trial that enrolled children who were on a drug of interest (e.g., ampicillin) and collected low-volume pharmacokinetics samples.^8^ Notably, POPS did not account for cerebral spinal fluid (CSF) penetration of ampicillin; thus, this research cannot be used to determine meningitic dosing. In 2019, the American Academy of Pediatrics (AAP) provided dosing recommendations for group B *Streptococcus* (GBS) bacteremia and meningitis based on POPS data and historic standard of care, respectively.^9^ Because GBS remains the most common gram-positive cause of early-onset neonatal sepsis, their recommendations guide empiric treatment of neonates. Notably, FDA ampicillin labeling has recently been updated, and its recommendation for neonatal bacteremia dosing now matches that of the AAP.^10^ However, unlike the AAP guidelines, FDA labeling does not recommend a higher dose for meningitis.

Significant variability in antibiotic dosing in children in European hospitals has been described, particularly in NICUs.^11,12^ This variability in dosing suggests suboptimal exposures for some patients. However, to our knowledge, dosing variability in ampicillin has not previously been studied in US children’s hospitals.

We examined variability in ampicillin dosing in pediatric hospitals across 3 common indications: (1) bacterial lower respiratory tract infections (LRTIs), (2) neonates (infants aged ≤28 days) with suspected or proven sepsis, and (3) neonates with suspected CNS infections. We hypothesized that daily dosage of ampicillin would vary within these indications and would not consistently adhere to available recommendations or guidelines. The purpose of our study was to identify variation in ampicillin dosing that could be targeted by future efforts in antibiotic stewardship.

## Methods

We performed our analysis using data from the SHARPS point prevalence study (PPS) of antimicrobial use, which was a serial, cross-sectional survey of antibiotic prescribing at 32 US acute-care children’s hospitals from July 2016 to December 2017.^2^ On a single day during 6 quarterly periods, antimicrobial stewardship program (ASP) members created a record of active antimicrobial prescriptions for inpatients with both patient specific data (including hospital service, gestational age, postnatal age, and weight) as well as antimicrobial data (route, dose, dosing interval, and indication), which were determined via chart review by physicians and/or pharmacists. Reviewers selected indications based on a predefined list of 25 grouped conditions and selected a single, primary indication for therapy.^2^ They also collected data regarding whether the ASP would routinely review the antibiotic based on individual ASP practices at participating institutions. Quality assurance procedures have been described in detail previously and included an e-learning training course, supplementary training documents, a help desk, and software support with warning messages for missing or potentially erroneous data.^13^

For this analysis, we included ampicillin PPS data with a recorded indication for (1) lower respiratory tract infections in infants and children >3 months, (2) neonates suspected or proven sepsis, and (3) neonates with probable or proven CNS infections. Additionally, prescriptions classified as “newborn prophylaxis for maternal risk factors” or “newborn prophylaxis for newborn risk factors” were included in the analysis of neonates with suspected or proven sepsis. We excluded prescriptions from the analysis if a weight was not provided or if a data entry error was identified. LRTI prescriptions were excluded if patients were based in the NICU or were aged <3 months or if the total daily dose was equivalent to the maximum recommended dose of 8 g per day. Neonatal prescriptions were excluded if patients were aged >28 days or did not have a documented gestational age despite admission to the NICU.

Prescriptions were analyzed for total daily dose per kilogram and presence of ASP review. Bacterial LRTI prescriptions were compared to the recommended IDSA uncomplicated CAP dosing of 150–200 mg/kg/day.^1^ Prescriptions for neonates with suspected or proven sepsis or meningitis were grouped according to estimated gestational age (EGA) and postnatal age and were compared to the AAP GBS dosing guidelines (Table 1).^8,9^ If neonates were not in the NICU, no gestational age was documented, and these infants were assumed to be >34 weeks EGA. Because EGAs were documented in weeks only (as opposed to weeks and days), neonates with a documented EGA of 34 weeks were also assumed to be >34 weeks EGA. Prescriptions were classified as suboptimal if they were <80% of recommended dosing, optimal if they were within 80%–120%, or excessive if they exceeded 120%. This stratification allowed a greater margin of dose rounding than the 10% utilized in most computerized physician order entry systems. Prescriptions for neonates were also graphically compared to current neonatal dosing recommendations in Neofax, the *Nelson Textbook of Pediatrics*, and *The Harriet Lane Handbook*.^5–7^

**Table 1.** Neonatal Dosing for Bacteremia and Meningitis per AAP GBS Guidelines by Estimated Gestational Age at Birth (EGA) and Postnatal Age (PNA)

EGA,weeks	PNA, days	Bacteremia			GBS Meningitis					
		mg/kg/dose	Dosing Interval, hours	mg/kg/day	mg/kg/dose	Dosing Interval, hours	mg/kg/day	No. of Prescriptions	Median Prescribed Dose, mg/kg/day	IQR
≤34	≤7	50	12	100	100	8	300	105	200	190.6–205.1
	8–28	75	12	150	75	6	300	9	175.6	170.2–210
>34	≤7	50	8	150	100	8	300	283	200	192.1–219.8
	8–28	50	8	150	75	6	300	102	206.6	198.7–300.6

Note. GBS, group B *Streptococcus*; IQR, interquartile range.

### Statistical analysis

A statistical analysis was performed, and figures were created using Microsoft Excel version 16.17 software (Microsoft, Redmond, WA). Prescriptions included for each indication were analyzed for range, median, and interquartile range (IQR). Box plots were created to graphically compare prescribed dosages to recommendations from the AAP, Neofax, the *Nelson Textbook of Pediatrics*, and *The Harriet Lane Handbook*.

### Study approval

The SHARPS PPS study was reviewed and approved by the institutional review board (IRB) at Children’s Mercy–Kansas City and/or through the IRB of the local institution.

## Results

### Lower respiratory tract infections in infants and children aged >3 months

In total, 1,038 ampicillin prescriptions from 32 hospitals were included in our analysis. Among them, 129 prescriptions were for proven or probable bacterial LRTI. For LRTI, prescriptions were excluded for the following reasons: no weight was listed (n = 4); the total ampicillin dose was equal to the maximum dose of 8 g per day (n = 14); the patient was in the NICU (n = 14); or the patient was admitted to a non-NICU service but was aged <3 months (n = 9). The remaining 88 prescriptions were included in the analysis for proven or probable bacterial LRTI. Total mg/kg/day ranged from 100.2 mg/kg/day to 402.1 mg/kg/day (median, 200; IQR, 197.2–209.7). When compared to the recommended IDSA LRTI dosing of 150–200 mg/kg/day, 1 (1.1%) was suboptimal, 68 (77.3%) were optimal, and 19 (21.6%) were excessive.

### Suspected or proven neonatal sepsis

**Fig. 1.**
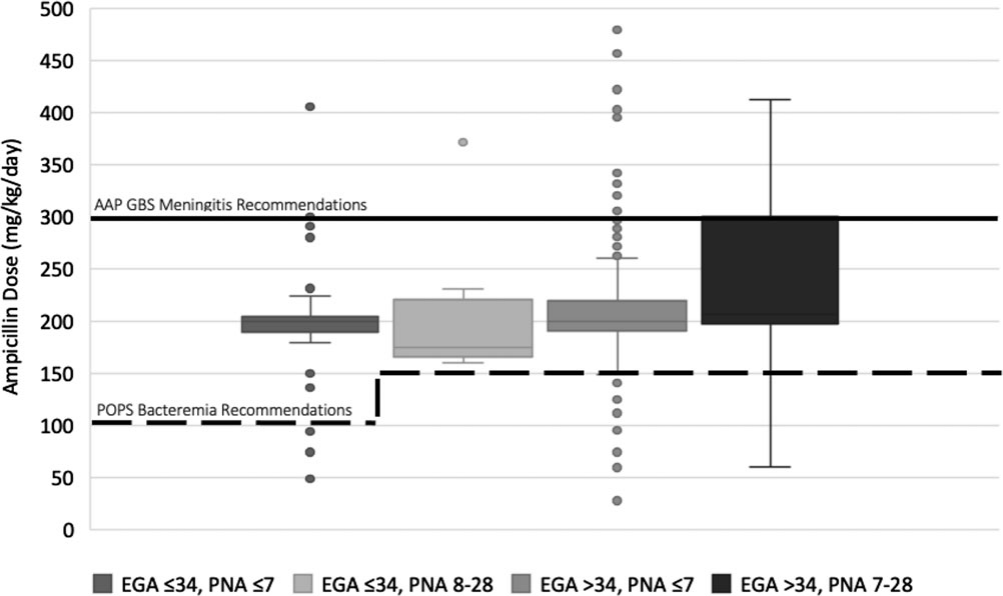
Ampicillin dosing for neonates with suspected or proven sepsis compared to American Academy of Pediatrics (AAP) recommendations. Dosing is in mg/kg/day, grouped by estimated gestational age at birth (EGA) and postnatal age (PNA), and is compared to AAP recommendations for bacteremia (dotted line) and AAP recommendations for group B *Streptococcus* (GBS) meningitis (solid line).

In total, 585 prescriptions were classified as being for suspected or proven sepsis in an infant. Prescriptions were excluded for the following reasons: age >28 days (n = 73), lacking a documented weight (n = 9), unknown NICU status (n = 2), presence in NICU with unknown gestational age (n = 1), and concern for erroneous data entry (n = 1). The remaining 499 prescriptions were included in the analysis for suspected/proven neonatal sepsis (Table 1 and Fig. 1).^8,9^ Total mg/kg/day ranged from 28.1 mg/kg/day to 480 mg/kg/day (median, 200; IQR, 193.1–219.4). Compared to recommended AAP dosing for neonatal bacteremia, 28 prescriptions (5.6%) were suboptimal, 64 (12.8%) were optimal, and 407 (81.6%) were excessive. Compared to GBS meningitis dosing recommended by the AAP, 393 (78.8%) were suboptimal, 89 (17.8%) were optimal, and 17 (3.4%) were excessive. Prescriptions were also graphically compared to current neonatal dosing recommendations in additional tertiary references (Supplementary Figs. 1–3). These figures highlight similar findings of generally excessive dosing but also the marked variability in empiric dosing recommendations for neonates.

### Suspected or proven neonatal CNS infections

Finally, dosing was examined in 27 neonates categorized as having a probable or proven CNS infection. The total mg/kg/day ranged from 98.4 to 404 (median, 270.8; IQR, 199.0–300.0). When compared to GBS meningitis dosing recommended by the AAP, 13 (48.1%) were suboptimal, 11 (40.7%) were optimal, and 3 (11.1%) were excessive.

### Antibiotic stewardship review

In terms of ASP review, 127 (20.7%) of LRTIs and neonatal prescriptions had already been reviewed by stewardship, 174 (28.3%) would reportedly be reviewed soon, and 311 (50.7%) would not have been reviewed (either due to lack of an ASP or because a review would not be routinely performed). Review status was not indicated for 2 prescriptions. Only in 20 (15.7%) reviewed prescriptions did reviewers recommend frequency or dose modification.

## Discussion

This study is the first to examine ampicillin dosing practices in US children’s hospitals for common indications including bacterial LRTI in pediatric patients and suspected or proven bacterial infections in neonates. Our research revealed that bacterial LRTI dosing is generally within the current range recommended by the IDSA. However, dosing for suspected or proven sepsis in neonates tended to be more variable and was often above the recommended dosing for bacteremia but below the recommended dosing for meningitis. Even neonates specifically thought to have a CNS infection did not uniformly receive ampicillin dosed in accordance with AAP recommendations for GBS meningitis. Finally, half of all ampicillin prescriptions were not reviewed by ASP teams, and when prescriptions were reviewed, dosing was rarely commented upon.

First, our review demonstrated that ∼75% of ampicillin prescriptions for LRTI are within the dosing range suggested by the IDSA. This is consistent with prior research suggesting that the 2011 IDSA CAP guidelines influenced antibiotic prescribing patterns.^14^ It follows logically that when dosing guidance is clear and widely disseminated, providers are more likely to prescribe consistent, optimal doses.

In contrast to the relative consistency of LRTI prescriptions, neonatal prescriptions are unlikely to be dosed in accordance with new evidence-based recommendations for bacteremia, with excessive dosing in 81% of prescriptions. One explanation could be that providers are administering higher doses to provide meningitic coverage; the POPS study did not account for CNS penetration. Neonatal meningitis is rare, occurring in 0.13–0.7 per 1,000 live births,^15,16^ thanks in large part to maternal screening and prophylaxis for group B *Streptococcus* colonization. However, neonatal bacterial meningitis can be devastating; contemporary mortality rates remain as high as 18%, and survivors have a high risk of serious neurologic sequelae.^17^ Thus, it may be challenging for providers to intentionally prescribe an antibiotic dose below those recommended for GBS meningitis, even when clinical concern is low.

However, 78.9% of neonatal doses in our study were >20% lower than the dosing recommended by the AAP for GBS meningitis, suggesting that providers are either following older tertiary recommendations, which are numerous, variable, and often challenging to interpret, or that providers are following institution-specific guidelines. Furthermore, even among neonates in whom a CNS infection was specifically suspected, almost 50% of prescriptions were below the AAP GBS meningitis recommendations. The lack of adherence to the AAP GBS guidelines raises concern. However, it should be noted that current meningitic dosing recommendations are not derived from robust pharmacokinetic studies. In addition, reduced renal function before 34 weeks necessitates consideration of lower doses for premature neonates, which is not reflected in the current AAP guideline for meningitis.^18^ Unfortunately, repeat lumbar punctures after initiation of antibiotics are rarely performed, making an opportunistic CNS penetration study modeled after POPS not feasible.

Finally, the lack of dosing guidance and, in just >50% of cases, total lack of stewardship review presents an opportunity for increased stewardship efforts, in this case, specifically in collaboration with clinical pharmacists serving the NICU and targeting dosing. One could argue that, given the general safety of ampicillin, this antibiotic is a lower-yield area of focus. However, no medication is without risk. In a large EHR review, higher ampicillin exposures in neonates were associated with increased risk of seizure.^19^ In addition, all excessive antimicrobial exposures carry the theoretical risks of disruption of the microbiome, emergence of multidrug resistance, and increased cost.

Our study had several limitations. We reviewed data from 2016–2017, only a few years after publication of the new POPS evidence-based dosing study and prior to publication of the AAP GBS guideline; adherence to their recommendations may have increased since this time. However, the tertiary references described in the supplementary figures continue to reflect historic dosing suggesting uptake in clinical practice remains incomplete.^5–7^ Data were also gathered at discrete points in time, which may not accurately reflect the dosing given for the full antibiotic course. Reviewers selected indications for prescriptions from a predefined list, which could result in misclassification. A standard operations manual was provided to increase consistency across sites. Additionally, data entry errors are also possible. Finally, follow-up data for patients in this study are unavailable, prohibiting determination of outcomes in this cohort.

A further important limitation is that deviation from general IDSA and AAP recommendations may at times be appropriate. For example, higher doses of ampicillin are indicated for LRTIs caused by resistant isolates of *Streptococcus pneumoniae*. In addition, some neonatal units may utilize estimates of neonatal renal maturation to inform lower dosing for very premature neonates with suspected meningitis, as is the case at our institution. Dose reduction may also be considered in cases of acute kidney injury. Finally, inability to rule out CNS infection may have appropriately led to higher dosing in some of the infants classified as having suspected or proven neonatal sepsis.

Despite these limitations, our findings suggest important opportunities for improving ampicillin dosing. When dosing recommendations are clear and widely disseminated, as is the case for LRTI dosing, most prescriptions fall within the recommended range. However, when multiple resources are available, as with neonatal dosing, provider choices are less likely to reflect evidence-based recommendations. Opportunities for improving neonatal ampicillin dosing include wider dissemination of evidence-based dosing recommendations for bacteremia, provision of clearer guidance for when bacteremic versus meningitic dosing should be used, and, if possible, further research into evidence-based meningitic dosing. Finally, the lack of current stewardship focus on ampicillin suggests an opportunity for increased stewardship interventions for ampicillin use in particular and antibiotic dosing in general.

## Supplementary material

For supplementary material accompanying this paper visit https://doi.org/10.1017/ash.2022.221. click here to view supplementary material
