# Supplementary material for: Variation in ampicillin dosing for lower respiratory tract infections and neonatal bacterial infections in US children’s hospitals
Source: Antimicrob Steward Healthc Epidemiol. 2022 May 23;2(1):e85. doi: 10.1017/ash.2022.221 (PMC9726562; doi:10.1017/ash.2022.221)
Supplement: Supplementary file 1 [file S2732494X22002212sup001.docx]

**List of Supplemental Digital Content:**

Supplemental Digital Content 1. Figure

Supplemental Digital Content 2. Figure

Supplemental Digital Content 3. Figure

**Supplemental Digital Content 1:**

Upper limit of NeoFax Recommendations

Ampicillin dosing for neonates with suspected/proven sepsis compared to NeoFax recommendations. Dosing is in mg/kg/day, grouped by estimated post-menstrual age (PMA) and postnatal age (PNA), and is compared to NeoFax recommendations for bacteremia (red line).

**Supplemental Digital Content 2:**

Nelson’s Recommendations

Ampicillin dosing for neonates with suspected/proven sepsis compared to Nelson’s recommendations. Dosing is in mg/kg/day, grouped by weight (kg) and postnatal age (PNA), and is compared to Nelson’s recommendations (red line).

**Supplemental Digital Content 3:**

Harriet Lane Recommendations

Ampicillin dosing for infants with suspected/proven sepsis compared to Harriet Lane recommendations. Dosing is in mg/kg/day, grouped by postnatal age (PNA) and weight (kg), and is compared to Harriet Lane bacteremia recommendations (red line).
